# A Novel Theanine Complex, Mg-L-Theanine Improves Sleep Quality *via* Regulating Brain Electrochemical Activity

**DOI:** 10.3389/fnut.2022.874254

**Published:** 2022-04-05

**Authors:** Muhammed Furkan Dasdelen, Sezgin Er, Berkan Kaplan, Suleyman Celik, Mustafa Caglar Beker, Cemal Orhan, Mehmet Tuzcu, Nurhan Sahin, Havakhanum Mamedova, Sarah Sylla, James Komorowski, Sara Perez Ojalvo, Kazim Sahin, Ertugrul Kilic

**Affiliations:** ^1^International School of Medicine, Istanbul Medipol University, Istanbul, Turkey; ^2^Department of Neurology, Istanbul Medipol University, Istanbul, Turkey; ^3^Department of Physiology, School of Medicine, Istanbul Medipol University, Istanbul, Turkey; ^4^Department of Animal Nutrition, Faculty of Veterinary Medicine, Firat University, Elazig, Turkey; ^5^Scientific and Regulatory Affairs, Nutrition21, LLC, Purchase, NY, United States

**Keywords:** L-theanine, magnesium, GABA receptors, neurotransmitters, sleep

## Abstract

L-Theanine is commonly used to improve sleep quality through inhibitory neurotransmitters. On the other hand, Mg^2+^, a natural NMDA antagonist and GABA agonist, has a critical role in sleep regulation. Using the caffeine-induced brain electrical activity model, here we investigated the potency of L-theanine and two novel Mg-L-theanine compounds with different magnesium concentrations on electrocorticography (ECoG) patterns, GABAergic and serotonergic receptor expressions, dopamine, serotonin, and melatonin levels. Furthermore, we evaluated the sleep latency and duration in the pentobarbital induced sleep model. We herein showed that L-theanine, particularly its various complexes with magnesium increases the expression of GABAergic, serotonergic, and glutamatergic receptors, which were associated with decreased ECoG frequency, increased amplitude, and enhanced delta wave powers. Besides increased dopamine, serotonin, and melatonin; decreased MDA and increased antioxidant enzyme levels were also observed particularly with Mg-complexes. Protein expression analyses also showed that Mg-L-theanine complexes decrease inducible nitric oxide synthase (iNOS) and endothelial nitric oxide synthase (eNOS) levels significantly. In accordance with these results, Mg complexes improved the sleep latency and duration even after caffeine administration. As a result, our data indicate that Mg-L-theanine compounds potentiate the effect of L-theanine on sleep by boosting slow-brain waves, regulating brain electrical activity, and increasing neurotransmitter and GABA receptor levels.

## Graphical Abstract

The effects of Mg-L-Theanine on brain electrochemical activity, antioxidant enzymes and sleep. Mg-L-Theanine increases sleep duration, slow waves powers, GABAergic and serotonergic receptor expressions, dopamine, melatonin, and serotonin levels, and antioxidant enzyme activity.

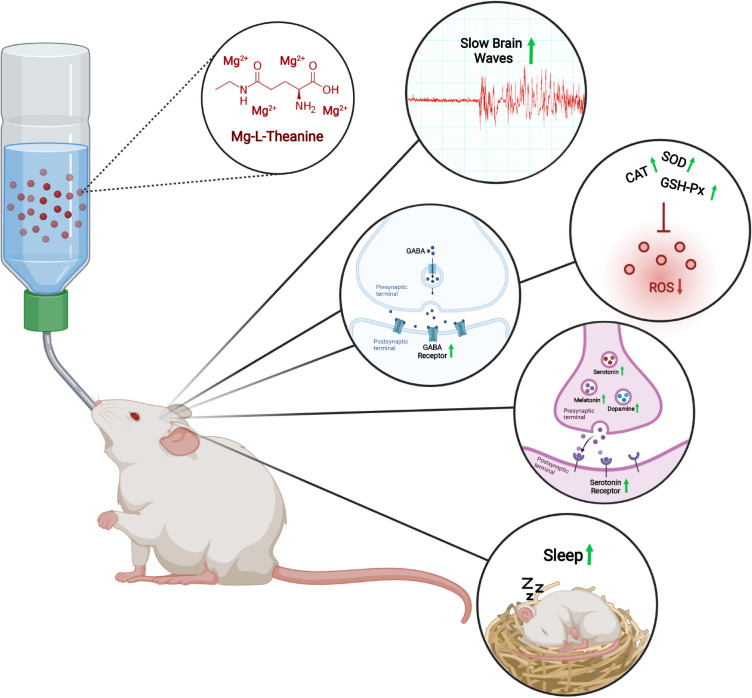

## Introduction

Sleep disturbances encompass numerous disorders which have detrimental impacts on the quality of life. Sleep problems, including difficulty in falling asleep, decreased sleep duration, and inconsistent sleep/wake patterns affect 56% of people in the United States, 31% in Western Europe, 23% in Japan (1), and are a significant cause of morbidity and mortality (2). Although the importance of having a good sleep is well-established, most people suffering from low-quality sleep do not look for medication since many hypnotics and sedatives have side effects. Hypnotic drugs such as benzodiazepines are associated with memory impairment, dementia, depression, and cause dependency and withdrawal phenomena in chronic usage (3). Despite non-benzodiazepine sedatives having lesser serious side effects than benzodiazepines, they have the potential to cause anterograde amnesia, headache, dizziness, and unpleasant taste (4–7). Hence, it is required to develop new therapeutic agents with lesser side effects but as effective as hypnotics, e.g., L-theanine and GABA.

L-Theanine, a non-proteinogenic amino acid particularly found in green tea, is a well-known agent for improving sleep disturbances (8, 9). It is structurally similar to the excitatory neurotransmitter glutamate in the brain and possibly blocks glutamate receptors in the central nervous system (10). It is commonly used to treat sleep disturbance, improve non-rapid eye movement (NREM) sleep, and reduce psychological stress. Previous studies suggest that L-theanine exerts its relaxant effect by enhancing GABA levels, thereby increasing the expression of dopamine and serotonin in the brain (11–13). In animal studies, L-theanine was shown to oppose caffeine’s effect and promote sedation (11, 14–16). In addition to its relaxant potency, L-theanine has a neuroprotective role since it acts as a glutamate receptor antagonist, upregulates GABA receptors, and increases the expression of antioxidant enzymes (17–20).

Apart from L-theanine, magnesium is also a glutamate receptor antagonist and may play a critical role in regulating sleep (21). Previous studies on rodents indicated that Mg^2+^ ions serve as a critical signal for synapse formation and increased intraneuronal Mg^2+^ concentration was associated with an increase in synaptic density and plasticity in the prefrontal cortex and hippocampus in young and old rats (22–24), as well as enhancement of short/long-term memory, reduction of anxiety, reduction in depression (25–27). Besides, increased Mg^2+^ *via* various substances was suggested to improve memory of patients with Alzheimer’s disease by inhibiting the neuroinflammation (22, 28, 29). Moreover, molecular and animal studies have shown that Mg^2+^ pretreatment shows neuroprotective effects (30, 31). Those neuroprotective effects were attributed to Mg^2+^’s antagonistic interaction with NR2B-containing NMDA receptors, and therefore prevention of excitotoxicity.

Magnesium L-theanine is a novel compound consisting of Mg^2+^ ions and L-theanine molecules. It readily passes to the blood–brain barrier, increases the Mg^2+^ ion levels of CSF, and has a potential sleep-regulating effect similar to L-theanine (32, 33). Although magnesium and L-theanine have numerous effects on CNS and mood-related disorders separately, there has been no research concerning the effects of Mg-L-theanine compounds on sleep and brain activity. Hence, in this study, we aimed to determine the potency of different Mg-L-theanine compounds with different magnesium concentrations on sleep latency and duration, brain electrical activity, neuronal activity, and antioxidant parameters compared to L-theanine in caffeine-induced sleep disturbance and pentobarbital induced sleep models in mice.

## Materials and Methods

### Experimental Animals and Design

**FIGURE 1:**
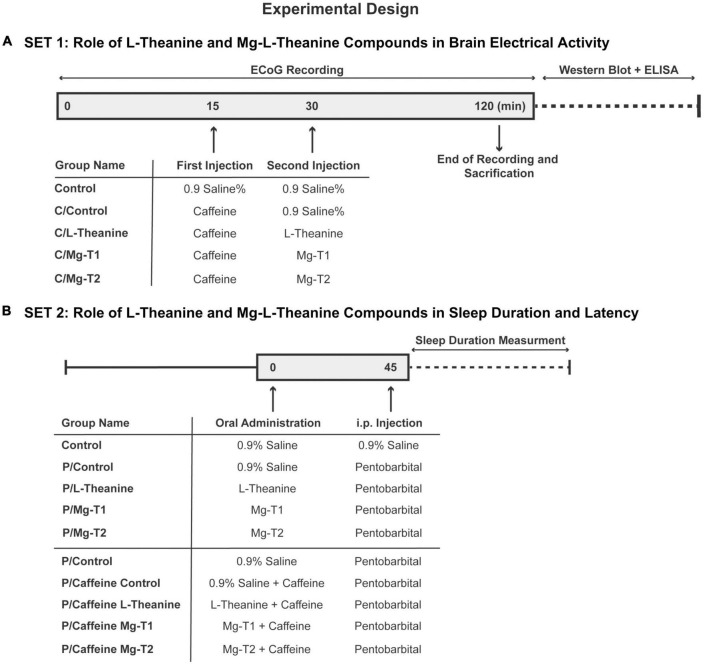
Experimental design. Experimental design and animal groups, *n* = 6 for the first set **(A)**, *n* = 8 for the second set **(B)**.

This study has been conducted under National Institutes of Health (NIH) guidelines for the care and use of laboratory animals and approved by local government authorities (Istanbul Medipol University, Animal Research Ethics Committee). All animals were maintained under a constant 12 h light/dark regimen (light on at 07.00 a.m. daily) in a temperature-controlled room (21 ± 1°C) with *ad libitum* access to food and water. Nine-week-old male Balb/c mice were randomized equally into two sets of experiments (Figure 1).

In the first set of experiments, animals were separated into one of the following groups (*n* = 6 per group): Control (0.9% saline followed by 0.9% saline), C/Control (caffeine followed by 0.9% saline), C/L-theanine (caffeine followed by L-theanine), C/Mg-T1 [caffeine followed by Magnesium-L-theanine (8% Mg^2+^)], or C/Mg-T2 [caffeine followed by Magnesium-L-theanine (18% Mg^2+^)]. Caffeine (7.5 mg/kg), L-theanine (20 mg/kg), Mg-T1 (21.74 mg/kg), and Mg-T2 (24.39 mg/kg) were dissolved in 0.9% saline and injected intraperitoneally. The amounts of L-theanine per kg of mice were equal in C/L-theanine, C/Mg-T1, and C/Mg-T2 groups (20 mg/kg). After 2 h of electrocorticography (ECoG) recording, animals were sacrificed and brains were used for the analysis of the levels of neurotransmitters, related neurotransmitter receptors, and antioxidant enzymes.

In the second set of experiments, the effect of Mg-L-theanine compounds with different magnesium concentrations compared to L-theanine on sleep quality was determined. Animals were divided into five groups: Control (0.9% saline followed by 0.9% saline), P/Control (pentobarbital followed by 0.9% saline), P/L-theanine (pentobarbital followed by L-theanine), P/MgT1 [pentobarbital followed by Magnesium-L-theanine (8% Mg^2+^)], or P/MgT2 (pentobarbital followed by Magnesium-L-theanine (18% Mg^2+^). Mg-T or L-theanine compounds were given at the same dose as the first experiment. Forty-five minutes following treatment, pentobarbital was given 30 mg/kg intraperitoneally after dissolved in 0.9% saline. Sleep duration, sleep latency and the number of animals that fall into sleep were compared among groups. The same sleep test was applied to the caffeine-induced insomnia model of mice. For this purpose, 50 mg/kg caffeine were added to oral regimens except Control group’s regimen and after 45 min, pentobarbital was administered to all groups for sleep evaluation.

### Induction of Sleep Disturbance and Electrocorticography Recording

Animals were anesthetized with urethane (1.25 g/kg, i.p., Sigma U2500) and carefully placed in a stereotaxic frame. Rectal temperature was maintained between 36.5 and 37.0°C using a feedback-controlled heating system (507221F, Harvard Apparatus, ABD). A midline incision was made on the skin along the sagittal suture of the skull. A part of the cranium overlying the left parietal cortex was removed using a dental drill. Ag–AgCl ball electrodes were placed on the left somatomotor cortex (1 mm anterior/1.5 mm lateral from bregma; 3 mm posterior/1.5 mm lateral from bregma) and the reference electrode was attached to the left foot. To induce sleep disturbance, 7.5 mg/kg caffeine was injected intraperitoneally at the 15th minute of recordings, and second injections (Saline, L-theanine, Mg-T1, or Mg-T2) were performed at the 30th minute according to groups. Brain electrical activity was monitored and recorded for a total of 2 h. Signals were sampled at 1000 Hz with a band-pass filter set at 0.5-500 Hz by using the PowerLab system (16/30, AD Instruments, Castle Hill, NSW, Australia). Raw data were stored for later offline analysis. The spike-frequency, amplitude, and power spectral analysis were performed using LabChart 8.1.17 software (ADInstruments, Bella Vista, NSW, Australia).

Animals were sacrificed under deep anesthesia after 2 h of ECoG recording. Brains were removed, frozen on dry ice, and stored at −80°C for subsequent Western blot and enzyme-linked immunosorbent assay (ELISA) experiments.

### Western Blot

Brain tissue samples were collected from each animal for Western blot analysis, and studies were carried out as described by Beker et al. (34). Briefly, tissue samples from the same group were pooled, homogenized, sonicated, and treated with a protease inhibitor cocktail and a phosphatase inhibitor cocktail. The total protein content was determined using the Qubit 2.0 Fluorometer, following the manufacturer’s instructions (Invitrogen, Life Technologies Corporation, Carlsbad, CA, United States). Using the Trans-Blot TurboTransfer System, equal amounts of protein (20 g) were size-fractionated using any-kD Mini-Protean TGX gel electrophoresis and then transferred to a nitrocellulose membrane (Bio-Rad, Life Sciences Research). Membranes were blocked for 1 h at room temperature in 5% non-fat milk in 50 mmol Tris-buffered saline containing 0.1% Tween (TBS-T; blocking solution). After membranes were washed in 50 mmol TBS-T, primary antibodies against GABA_*A*_-R (Cat: ab92747, Abcam, Cambridge, United Kingdom), GABA_*B*_-R1 (Cat: PA5-27725, Thermo Fisher Scientific), GABA_*B*_-R2 (Cat: ab52248, Abcam, Cambridge, United Kingdom), 5-HT_1A_ (Cat: ab85615, Abcam, Cambridge, United Kingdom), GluA1 (Cat: ab31232, Abcam, Cambridge, United Kingdom), GluN1 (Cat: ab17345, Abcam, Cambridge, United Kingdom), Glun2A (Cat: ab203197, Abcam, Cambridge, United Kingdom), endothelial nitric oxide synthase (eNOS) (Cat: ab5589, Abcam, Cambridge, United Kingdom), and inducible nitric oxide synthase (iNOS) (Cat: ab178945, Abcam, Cambridge, United Kingdom) were added for overnight incubation. The next day, membranes were washed with 50 mM TBS-T and incubated for 1 h at room temperature with horseradish peroxidase-conjugated goat-anti-rabbit antibody (7074, Cell Signaling Technology, ABD). After stripping and reprobing, polyclonal rabbit anti-β-actin antibody were used to control protein loading (4967; Cell Signaling Technology). The Clarity Western ECL Substrate kit (Bio-Rad; Life Sciences Research) was used to generate the blots, which were then visualized using the ChemiDoc MP System (Bio-Rad; Life Sciences Research). Blot experiments were conducted at least three times to prevent technical errors. Protein levels were densitometrically assessed with the ImageJ tool and expressed as percent relative to the control group after all blots were corrected with β-actin values.

### Enzyme-Linked Immunosorbent Assay

Enzyme-linked immunosorbent assay kits from BT-LABS (EO219Ra, range: 0.02–6 ng/ml; sensitivity: 0.012 ng/ml; intra-assay: CV <8%; inter-assay: CV <10% for dopamine; EO866Ra, range: 0.5–200 ng/ml, sensitivity: 0.23 ng/ml, intra-assay: CV <8%; inter-assay: CV <10% for serotonin and EO120Mo for melatonin, range: 0.1–40 ng/ml, sensitivity: 0.015 ng/ml, intra-assay: CV <8%; inter-assay: CV <10%; Shanghai, China) were used to determine levels of dopamine, serotonin, and melatonin. Samples were lysed and homogenized in PBS with a glass homogenizer on ice. Dopamine, serotonin, and melatonin amounts were determined by using microplate reader at 450 ± 10 nm (Elx-800, Bio-Tek Instruments Inc., Winooski, VT, United States).

### Detection of Antioxidant Enzymes

Activities of superoxide dismutase (SOD), catalase (CAT), and glutathione peroxidase (GPx) were measured using the commercially available kits (BT-LABS, Shanghai, China) according to the manufacturer’s procedure. Sensitivities were 3.04 ng/ml, 052 ng/ml, and 2.21 U/ml for SOD, CAT, and GPx, respectively, and intra- and inter-assays were CV <8% and CV <10% for all. For MDA analyses, an HPLC apparatus of Shimadzu UV–vis SPD-10 AVP detector, a CTO-10 AS VP column, and 30 mM KH_2_PO_4_ and methanol (82.5: 17.5, v/v, pH 3.6) at a flow rate of 1.2 ml/min were used (Shimadzu, Japan). Column waste was monitored at 250 nm. For antioxidant enzymes and MDA analyses, tissue samples were rinsed in PBS and minced and homogenized in PBS with a glass homogenizer on ice, then thawed at 2–8°C and centrifuged at 2000–3000 rpm for 20 min.

### Induction of Sleep and Sleep Quality Evaluation

In the second set of experiments, animals received 20 mg/kg L-theanine, 21.74 mg/kg Magnesium-L-theanine (8% Mg^2+^), or 24.39 mg/kg Magnesium-L-theanine (18% Mg^2+^). To understand the effects of different forms of L-theanine on sleep quality, 42 mg/kg pentobarbital was administered intraperitoneally 45 min after treatments. Subsequently, mice were placed in cages individually and subjected to measurement of sleep duration and latency. Mice were considered to be asleep when they lost their righting reflex, which was defined as a failure of the mouse to right itself after being placed on its back. Time elapsed between pentobarbital injection and sleep onset was recorded as sleep latency. Sleep duration was defined as the time required for a mouse to recover after sleep onset.

### Statistics

For statistical data analysis, GraphPad Prism Software (GraphPad Software Inc., San Diego, CA, United States) was used. Differences between groups were compared with one-way ANOVA followed by LSD or Tukey’s HSD test and repeated measurements ANOVA. Values are given as mean ± SEM or ± SD. *p*-Values less than 0.05 were considered significant throughout the study.

## Results

### Effect of L-Theanine and Mg-L-Theanine Compounds on Brain Electrical Activity

**FIGURE 2:**
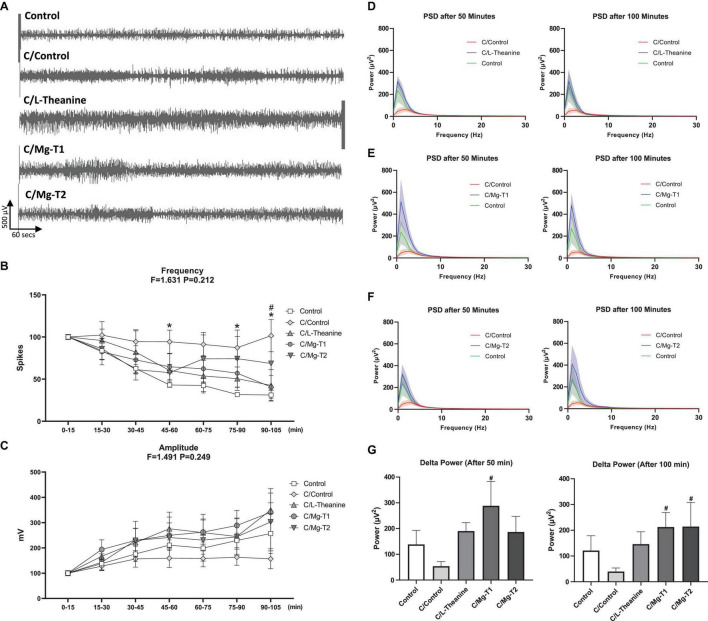
Effect of L-theanine and Mg-L-theanine compounds on brain electrical activity. Representative ECoG recordings for each group **(A)** and analyses of spike frequency **(B)** and amplitude **(C)** on ECoG. Values are represented as mean ± SEM for each group, *n* = 6. Symbols indicate significance by Fisher’s LSD as *p < 0.05 between Control and C/Control, ^#^*p* < 0.05 between C/Control and C/Mg-T1 groups. Frequency-power graphs of L-theanine **(D)**, Mg-T1 **(E)**, and Mg-T2 **(F)** at 50th and 100th minutes for 0–30 Hz range show restoration of slow brain waves after caffeine-induced decline which is then separately graphed for comparison of delta power between groups **(G)**. Average of the 1200 FFTs (10 min following 50th and 100th minutes) were calculated for each animal. ^#^*p* < 0.05 compared to C/Control group. All values are represented as mean ± SEM.

In the first set of experiments, efficacies of L-theanine and Mg-L-theanine compounds on brain electrical activity were compared (Figure 2). In all animal groups except C/Control, spike frequencies dropped as time elapsed. The spike frequencies were the highest, but amplitudes were the lowest throughout the recording in the C/Control group. The number of spikeswas significantly different at 45- to 60-, 75- to 90-, and 90- to 105-time intervals between Control and C/Control groups (*p* < 0.05). At 90–105-time intervals, spike frequencies were significantly lower in the C/Mg-T1 group compared to the C/Control (*p* < 0.05). Spike amplitudes of C/Mg-T1, C/Mg-T2, and C/ L-theanine groups were higher throughout the recording compared to C/Control, although no significant difference was observed.

### Mg-L-Theanine Treatments Restore Slow Brain Wave Reduction After Caffeine Injection

To understand the role of L-theanine and Mg-L-theanine compounds on brain waves, we performed power spectral density (PSD) analysis on ECoG recording. Brain wave frequencies between 0 and 30 Hz were examined and shown in Figures 2D–F. According to PSD assessments, caffeine injection decreases the power of delta waves. However, that effect was significantly reversed in the C/Mg-T1 group at 50th and 100th minutes (Figure 2G, *p* < 0.05). Moreover, we observed that delta waves were significantly enhanced in the C/Mg-T2 group at the 100th minute compared to the caffeine control group (Figure 2G, *p* < 0.05). No significant difference was observed on higher brain waves (Supplementary Figure 1).

### L-Theanine and Mg-L-Theanine Compounds Reverse Caffeine’s Effect on Inhibitory Receptors

**FIGURE 3:**
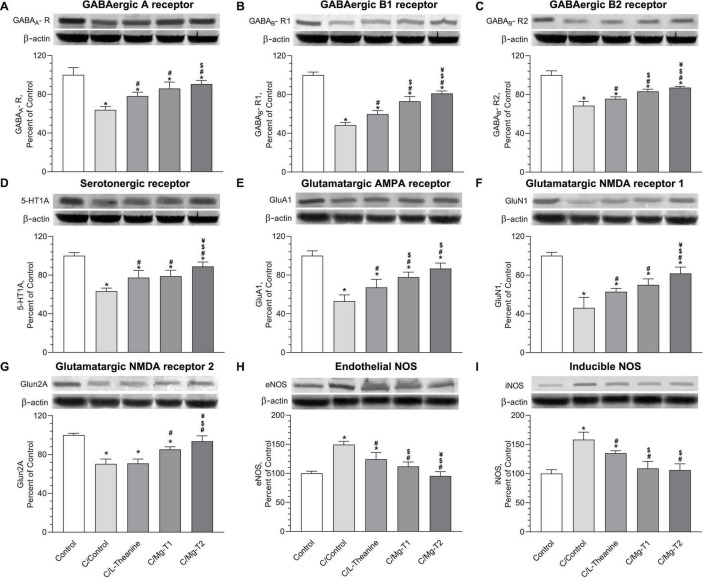
L-Theanine and Mg-L-theanine compounds reverse caffeine’s effect on inhibitory and glutamate receptors. Western blot results of GABAergic receptors GABA_*A*_-R **(A)**, GABA_*B*_-R1 **(B)**, GABA_*B*_-R2 **(C)**, serotonergic receptor 5-HT_1A_ **(D)**, glutamatergic receptors GluA1 **(E)**, GluN1 **(F)**, GluN2A **(G)**, and nitric oxide synthases, eNOS **(H)**, and iNOS **(I)**. All groups normalized according to housekeeping protein β-actin (three β-actin blots were performed for GABAergic and serotonergic receptors; glutamatergic receptors; and NOS), and one-way ANOVA with Tukey’s HSD was performed for multiple comparisons. Values are represented as mean ± SD. *p < 0.05: compared to control, ^#^*p* < 0.05: compared to caffeine control, ^$^*p* < 0.05: compared to L-theanine, ^¥^*p* < 0.05: compared to Mg-T1.

To examine the effects of L-theanine and Mg-L-theanine compounds on the inhibitory receptors GABA_*A*_-R, GABA_*B*_-R1, GABA_*B*_-R2, and 5-HT1A, we analyzed brain lysates from the five groups with Western blotting. Expression levels of those inhibitory receptors were evaluated and summarized in Figure 3. We measured reduced receptor levels in all experimental groups compared to the control group (*p* < 0.05). However, L-theanine and Mg-L-theanine compounds enhanced the levels of inhibitory receptors compared to the C/Control group (*p* < 0.05), by which they reversed caffeine-induced alterations on inhibitory receptors. We observed a significantly higher effect on the GABA_*A*_-R levels (Figure 3A) in the C/Mg-T2 group than the C/L-theanine group (*p* < 0.05), although we did not observe any significant difference between C/L-theanine and C/Mg-T1 groups. Moreover, we observed a higher increasing effect on the GABA_*B*_-R1 and GABA_*B*_-R2 levels both in C/Mg-T1 and C/Mg-T2 groups compared to C/L-theanine group (Figures 3B,C) (*p* < 0.05), and C/Mg-T2 group showed an even higher elevation than C/Mg-T1 group (*p* < 0.05). In addition, 5-HT_1A_ levels were significantly higher in the C/Mg-T2 group compared to C/Mg-T1 and C/L-theanine groups (Figure 3D) (*p* < 0.05).

### L-Theanine and Mg-L-Theanine Compounds Reverse Caffeine’s Effect on Glutamate Receptors

To elucidate the effects of L-theanine and Mg-L-theanine on the glutamatergic receptors, we measured glutamatergic AMPA receptor GluA1, and glutamatergic NMDA receptors GluN1, and GluN2a levels with Western blotting. Expression levels of those glutamate receptor subunits were evaluated and summarized in Figures 3E–G. According to the Western blot results, significantly reduced expressions of glutamate receptors were observed in all experimental groups compared to the control group (*p* < 0.05). Nevertheless, L-theanine and Mg-L-theanine administrations significantly increased the level of glutamate receptors compared to the C/Control group (*p* < 0.05), except, L-theanine did not cause a significant change in GluN2A levels. Moreover, in the C/Mg-T1 and C/Mg-T2 groups, we observed a greater influence on the GluA1 receptor levels compared to the C/L-theanine group (*p* < 0.05), while there was no significant difference between the two of them. Additionally, C/Mg-T2 group showed a higher GluN1 level than C/L-theanine and C/Mg-T1 groups (*p* < 0.05), while there was no significant difference between C/L-theanine and C/Mg-T1 groups. In addition, Mg-T1 and Mg-T2 significantly increased GluN2a levels in comparison to the C/Control group (*p* < 0.05), while in Mg-T2 the effect was even higher than Mg-T1 (*p* < 0.05).

To identify the effects of L-theanine and Mg-L-theanine on nitric oxide production, we analyzed the levels of oxidative stress markers eNOS and iNOS in the five groups with Western blotting. Expression levels of those factors were evaluated and summarized in Figures 3H,I. According to the results, eNOS, and iNOS were lower in the other three groups than the C/Control group (*p* < 0.05). However, no significant differences were observed between C/Mg-T1 and control groups and between C/Mg-T2 and control groups for eNOS levels, whereas eNOS levels were significantly lower in the C/Mg-T2 group compared to the C/Mg-T1 group (*p* < 0.05). As expected, the effects of the compounds on iNOS levels were similar to the eNOS levels except for the same expression levels of iNOS in C/Mg-T1 and C/Mg-T2 groups.

### L-Theanine and Mg-L-Theanine Compounds Exhibit Antioxidant Effects

**FIGURE 4:**
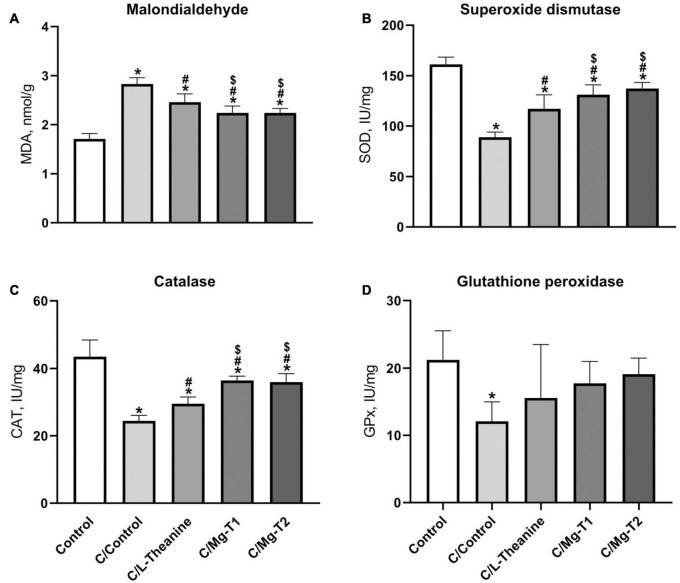
L-Theanine and Mg-L-theanine compounds exhibit antioxidant effects. MDA **(A)**, SOD **(B)**, CAT **(C)**, and GPx **(D)** levels in the brain. One-way ANOVA with Tukey’s HSD was performed for multiple comparisons and values are represented as mean ± SD. *p < 0.05: compared to control, ^#^*p* < 0.05: compared to caffeine control, ^$^*p* < 0.05: compared to L-theanine.

To identify the effects of L-theanine and Mg-L-theanine on antioxidation mechanisms, we performed ELISA on the five groups for malondialdehyde (MAD), SOD, CAT, and GPx. The levels of these markers were analyzed and summarized in Figure 4. All groups had lower SOD and CAT levels, whereas they had higher MDA levels than control (*p* < 0.05). However, C/L-theanine, C/Mg-T1, and C/Mg-T2 groups had higher SOD and CAT levels than the C/Control group (*p* < 0.05), while C/Mg-T1 and C/Mg-T2 groups showed even higher SOD and CAT levels (*p* < 0.05). Similarly, a reversal effect was observed for MDA as the MDA levels were lower after L-theanine, and Mg-L-theanine administrations (*p* < 0.05), where Mg-L-theanine compounds had higher decreasing effects than L-theanine (*p* < 0.05). We observed only one significant difference in GPx levels which was the decreased caffeine expression compared to the control group (*p* < 0.05).

### L-Theanine and Mg-L-Theanine Compounds Increase Sedative Neurotransmitter Levels in Caffeine Induced Insomnia

**FIGURE 5:**
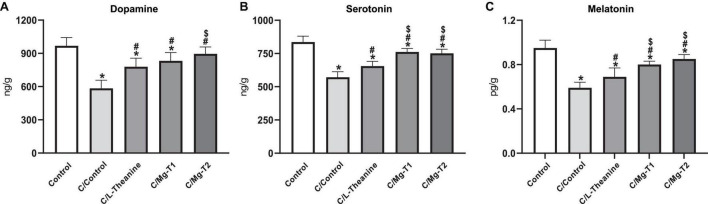
L-Theanine and Mg-L-theanine compounds improve sedative neurotransmitters. Dopamine **(A)**, serotonin **(B)**, and melatonin **(C)** levels in the brain. One-way ANOVA with Tukey’s HSD was performed for multiple comparisons and values are represented as mean ± SD. *p < 0.05: compared to control, ^#^*p* < 0.05: compared to caffeine control, ^$^*p* < 0.05: compared to L-theanine.

We next measured the effects of those compounds on the sedative neurotransmitters, dopamine, serotonin, and melatonin, with ELISA (enzyme-linked immunosorbent assay). Levels of the neurotransmitters were analyzed and summarized in Figure 5. They were significantly lower in all experimental groups than the control group (*p* < 0.05), excluding the dopamine level of C/Mg-T2 group, where no significant difference was observed. However, similar to our observation on inhibitory receptors, L-theanine and Mg-L-theanine compounds had increasing effects on the levels of the neurotransmitters compared to the C/Control group (*p* < 0.05). Furthermore, the C/Mg-T2 group showed a higher elevation of dopamine levels than the C/L-theanine and C/Mg-T1 groups (*p* < 0.05). Additionally, Mg-L-theanine administrations caused a higher increase in serotonin and melatonin levels compared to L-theanine (*p* < 0.05), while the difference between Mg^2+^ concentrations was insignificant.

### L-Theanine and Mg-L-Theanine Compounds Increase Sleep Duration and Decrease Sleep Latency After Pentobarbital Administration

**FIGURE 6:**
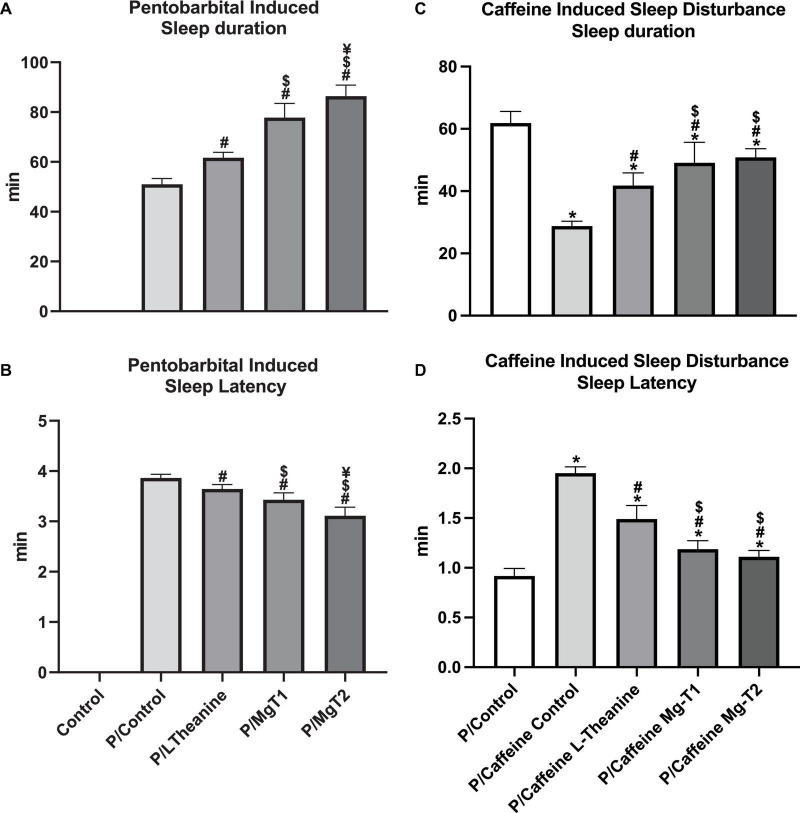
L-Theanine and Mg-L-theanine compounds increase sleep duration and de-crease sleep latency. Effects of L-theanine and Mg-T compounds on sleep duration **(A)** and sleep latency **(B)** after pentobarbital administration. The same test was applied in the caffeine-induced sleep disturbance model **(C,D)**. ANOVA with Tukey’s HSD was performed for multiple comparisons and values are represented as mean ± SD. *p < 0.05: compared to control, ^#^*p* < 0.05: compared to caffeine control, ^$^*p* < 0.05: compared to L-theanine, ^¥^*p* < 0.05: compared to Mg-T1.

In the second set of the experiments, to identify the effects of L-theanine and Mg-L-theanine on sleep, we measured the sleep duration and sleep latency of the mice in five groups after pentobarbital administration. The results of these experiments are shown in Figure 6. As expected, L-theanine and Mg-L-theanine compounds have increased the sleep duration and decreased the sleep latency compared to the P-Control group (*p* < 0.05), while in the P/Mg-T2 group, the effect was the highest (*p* < 0.05) and in the P/L-theanine group the effect was the lowest.

### L-Theanine and Mg-L-Theanine Compounds Attenuates Caffeine-Induced Sleep Disturbance

In order to understand the hypnotic effects of Mg-T compounds on sleep disturbance, we investigated whether different forms of L-theanine could enhance sleep quality and diminish insomnia triggered by caffeine administration. As shown in Figure 6, caffeine increases sleep latency and decreases sleep duration (*p* < 0.05). However, Mg-T and L-theanine molecules significantly reverse caffeine’s effect (*p* < 0.05). Although Mg-T compounds have significantly higher activity in terms of increasing the sleep duration and decreasing the sleep latency compared to L-theanine (*p* < 0.05), no difference was observed between Mg-T1 and Mg-T2.

## Discussion

Based on the previous research showing the improvement of L-theanine on sleep disturbance and its neuroprotective role, we examined the effects of a novel anxiolytic and cognition-enhancing agent Mg-L-theanine with different Mg^2+^ concentrations on sleep quality and brain chemical activity in comparison with L-theanine. We first analyzed the ECoG recordings to evaluate the brain waves, spike frequency, and spike amplitude. Although we did not see a general improvement in spike frequency and spike amplitude with L-theanine or Mg-T compounds on the caffeine-induced sleep disturbance model, we observed that in the Mg-T1 group, there had been a significant reduction in spike frequency after 90 min of recordings. According to PSD analyses, Mg-L-theanine with higher Mg^2+^ concentration has significantly boosted the delta waves at the 50th minute, and both Mg-L-theanine compounds have exhibited the same enhancing role at the 100th minute. Sleep is broken down into N1, N2, N3, and R stages (35), where the delta waves are high-amplitude slow brain waves, seen in N2 and N3, which correlates with sleep intensity in humans (36). Likewise, delta waves dominate NREM sleep in rodents (37) and are shown to be very important for memory consolidation by reactivating encoded neuronal memory and promoting their transference into long-term memory (38). Therefore, the enhancing effects of Mg-L-theanine compounds on delta waves may resolve insomnia, possibly intensify deep sleep stages, and regulate synaptic networks. We also performed a similar analysis for higher brain waves, in which we did not see a significant outcome. In contrast to the literature, we could not observe an improvement of L-theanine on the alpha power in our model, in which the mice were anesthetized with urethane and the recording electrodes were placed on the somatosensory cortex.

We next examined the levels of different inhibitory receptors, glutamate receptors, and sedative neurotransmitters, which are the key components of sleep homeostasis. GABAergic neurons are the main inhibitory neurons in sleep-inducing neural pathways, which naturally regulate wake-promoting circuitry (39). There are three types of GABA receptors, GABA_*A*_, GABA_*B*_, and GABA_*C*_. GABA_*A*_ receptors are fast-acting ligand-gated ion channels (40), which are the most abundant receptors seen in GABAergic neurons. They are well-known for regulating sleep-inducing circuits and are targeted to treat sleep-related problems. In fact, most sedative-hypnotic drugs (barbiturates and benzodiazepines) act through the GABA_*A*_ receptors (41). GABA_*B*_ receptors, on the other hand, are G protein-coupled receptors that mediate slow and prolonged inhibitory action through indirectly activating K^+^ channels, inactivating Ca^2+^ channels, and decreasing cyclic adenosine monophosphate (cAMP) levels (42). They are found to be a target for sleep problems in schizophrenia since GABA_*B*_ receptor agonists induce slow-wave sleep (SWS), although they have minimal impact on (rapid-eye movement) REM sleep (43). Herein, we found that caffeine induces insomnia through inhibition of GABA_*A*_ and GABA_*B*_ receptors while L-theanine and Mg-T compounds increase their expressions. In literature, L-theanine has been shown to stimulate GABA_*A*_ receptors directly (18) and indirectly by increasing the levels of GABA and thereby opposing caffeine’s effect (44). Likewise, certain concentrations of Mg^2+^ potentiate the effect of GABA on GABA_*A*_ receptors (45). We found that Mg-T compounds are more prominent in terms of increasing the level of GABA receptors, thereby promoting sleep, compared to L-theanine. Higher GABA receptor levels with Mg-T compounds can be explained by a synergistic effect of Mg^2+^ ions and L-theanine molecules. Furthermore, we observed the inhibitory serotonin receptor 5-HT_1A_ levels were increased with Mg-T compounds after the caffeine-induced decline. As shown in previous studies, 5-HT_1A_ receptors have a complex regulatory role on sleep. Their activation promotes waking status, induces SWS, or stimulates REM sleep depending on where 5-HT_1A_ receptors are located (46). Besides, serotonergic system dysfunctionality – reduction in synthesis, release, and metabolism of 5-HT and reduction in pre/postsynaptic density of 5-HT_1A_ receptors – mainly associated with mood and anxiety disorders (47). Thus, activation of the receptor by agonists shows an anxiolytic effect as well as an increase in SWS depending on the dose and location of delivery (48, 49). This sleep-inducing effect is further attributed to the activation of inhibitory 5-HT_1A_ autoreceptors located in Dorsal Raphe Nuclei (49). Hence, based on the other findings in our study, Mg-T compounds possibly induce sleep and reduce anxiety through inhibitory serotonin receptors as well as GABA receptors.

Animal studies suggest that L-theanine administration increases brain serotonin and dopamine levels (50). Concordantly, we observed that Mg-T and L-theanine administrations restore serotonin, dopamine as well as melatonin levels after caffeine-induced decline while higher activity belongs to Mg-T compounds. Melatonin is a naturally produced hormone by the pineal gland, regulates the sleep-wake cycle, and improves sleep quality, onset, and duration. Therefore, with its sleep-regulatory role, melatonin is frequently used as a therapeutic for insomnia in clinics. As Mg-T compounds increase melatonin levels in the brain, they may be used for the treatment of insomnia. Serotonin, on the other hand, is the precursor of melatonin, indicating a possible sleep-inducing role by increasing melatonin levels and activating inhibitory neurons. Blocking the synthesis of serotonin has been shown to reduce SWS in rats while administration of 5-HTP or L-Tryptophan restores the sleep back (51). We also observed an increase in dopamine levels which is mediated by stimulation of NMDA receptors (33), while its effect on sleep remains unclear, although in previous research, dopamine fluctuations were shown during sleep, and a peak in dopamine release was seen to occur just after sleep onset during the light phase (52).

Conversely, glutamate receptors are the main excitatory receptors in the central nervous system (53). Recent data have shown that those receptors may have a role in sleep regulation. Miracca et al. (54) showed that the deletion of the GluN1 NMDA receptor subunits creates highly inconsistent sleep-wake patterns and dampen REM sleep, indicating insomnia. They also pointed out that the NMDA glutamate receptor signaling is important in the firing of GABAergic sleep-related neurons. Here, consistent with the prior research, we observed that caffeine-induced sleep deprivation significantly decreases the GluN1, GluN2a, and GluN2b levels. However, Mg-T compounds together with L-theanine ameliorated those subunit levels. Previous studies suggest that L-theanine improves cognitive functions by direct affinity to AMPA and NMDA (55), mediating serotonin and dopamine release (50), and indirectly relieving stress and anxiety as we showed in this study. Therefore, increased levels of NMDA and AMPA receptor subunits both after Mg-T and L-theanine administrations support GABA receptors’ sleep-inducing activity.

In order to confirm our findings on receptor and neurotransmitter level alterations which were shown to be effective on sleep regulation, we performed a sleep induction test by pentobarbital administration after compounds were given with or without caffeine. Parallel to the previous studies (11), L-theanine not only reduced the sleep latency but also prolonged the sleep duration in the pentobarbital-induced sleep model. Besides, the Mg-T2 compound had the highest activity on increasing sleep duration and decreasing the time required to fall asleep. Moreover, caffeine reduces sleep duration and increases sleep latency of pentobarbital induced sleep. However, Mg-T compounds alleviate caffeine’s effect with the same efficacy. These results show that Mg-T compounds are potential molecules to increase the sleep quality of individuals who has insomnia and may diminish the side effects of pentobarbital or other hypnotic drugs.

In this study, apart from the sleep induction potencies, we also examined the effects of L-theanine and Mg-T compounds on antioxidant enzymes. Previous studies pointed out that antioxidant enzymes such as SOD, CAT, and GPx are crucial for protecting mitochondria from oxidative stress since the production of reactive species leads to mitochondrial damage, which is a key factor in the occurrence of neurodegenerative diseases (56). We found that L-theanine increases the activity of SOD, CAT but not GPx, which are similar to the findings of Zeng et al. (57). Zeng et al. indicate that L-theanine creates a significant difference in rats’ GPx levels at high doses (400 mg/kg) after brain damage. Moreover, we observed that Mg-T molecules increase the level of SOD and CAT after caffeine’s decreasing effect higher than L-theanine while they have a similar effect on GPx. In contrast to antioxidant enzyme activities, MDA, a highly active oxidative stress marker, decreases with L-theanine and Mg-T compounds while the highest effect belongs to Mg-T2 both on antioxidant enzymes and MDA levels. Thus, Mg-T molecules may exert neuroprotection by increasing the antioxidants after an oxidative stress condition such as cerebral ischemia. As shown in previous studies (58, 59), increased levels of melatonin and serotonin with Mg-T administration could further contribute antioxidant effect of novel L-theanine forms since those neurotransmitters decrease oxidative stress and play an important role in reactive oxygen species scavenging. However, a detailed study should be conducted on the protective role of Mg-T on neurodegeneration.

According to previous research, caffeine was shown to increase Ca^2+^ concentration in the vascular smooth muscle cells (VSMCs) through the cAMP pathway (60), by which it was found to increase the eNOS levels. Siamwala et al. (61) have also shown that L-theanine induces eNOS phosphorylation and increases nitric oxide (NO) production in endothelial cells, indicating possible protection against cerebrovascular diseases. Consistent with the literature, we observed an increase in eNOS levels after caffeine administration. However, contradicting the previous data, a decrease in eNOS levels was detected after L-theanine and Mg-T administrations. It is also indicated that caffeine increases the iNOS levels under basal conditions, whereas it decreases the iNOS expression after induction of inflammation by lipopolysaccharide (LPS) and IFN-γ (62, 63). Here, we observed that iNOS levels were enhanced after sole caffeine injection, similar to the previous results, while L-theanine and Mg-T injection after caffeine administration recovered that effect and reversed the increased iNOS levels. Given that hypoxic conditions such as stroke induce synthesis of NO by iNOS and this leads to exacerbation of the injury (64), Mg-L-theanine compounds may help reduction of inflammation by their anti-inflammatory role.

## Conclusion

In conclusion, our present study shows that L-theanine reverses the effects of caffeine on sleep disturbance, sleep disturbance-related brain chemistry alterations, and the caffeine’s effects on oxidative stress, where L-theanine seems to protect against neurodegenerative disorders. Our data further indicate that Mg-L-theanine compounds with different Mg^2+^ ratios, novel L-theanine agents, show even better improvement on sleep disturbance and sleep disturbance-related neurochemical changes. Therefore, here we provided a possible neurochemical mechanism of the Mg-T compounds that are responsible for their sleep-inducing role. Furthermore, we showed the antioxidant characteristics of Mg-L-theanine molecules which can be helpful to prevent acute neurodegenerative disorders. In addition, in the present study, to visualize and analyze the effects of studied compounds on brain spike activity, we conducted ECoG recordings by placing two Ag/Ag-Cl electrodes directly on the cortex while mice were under anesthesia. The reason we have chosen to record animals under anesthesia was to ensure that data are not affected by movements and myoelectrical artifacts and the allowance of urethane for prolonged recordings. Thus, continuous EEG recording can be conducted to understand the effect of Mg-T molecules on brain waves while mice are falling asleep in more physiological conditions and under influence of circadian rhythm.

## Data Availability Statement

The original contributions presented in the study are included in the article/Supplementary Material, further inquiries can be directed to the corresponding author.

## Ethics Statement

The animal study was reviewed and approved by the Istanbul Medipol University, Animal Research Ethics Committee.

## Author Contributions

MD, KS, and EK designed all the experiments. MD, SE, BK, SC, and HM carried out the animal experiments and performed the statistical analysis. MB, CO, MT, and NS conducted the Western blot and ELISA experiments. SS, JK, SO, KS, and EK executed the compound synthesis. EK, KS, and SE prepared the manuscript. All authors contributed to the article and approved the submitted version.

## Conflict of Interest

SS, JK, and SO were employed by Nutrition21, LLC. The remaining authors declare that the research was conducted in the absence of any commercial or financial relationships that could be construed as a potential conflict of interest.

## Publisher’s Note

All claims expressed in this article are solely those of the authors and do not necessarily represent those of their affiliated organizations, or those of the publisher, the editors and the reviewers. Any product that may be evaluated in this article, or claim that may be made by its manufacturer, is not guaranteed or endorsed by the publisher.

## Supplementary Material

Supplementary Figure 1 Frequency – power graphs of L-theanine **(A)**, Mg-T1 **(B)**, and Mg-T2 **(C)** at 50th and 100th minutes for 30–100 Hz range. Average of the 1200 FFT (10 min following 50th and 100th minutes) results were calculated for each animal and data were represented as mean ± SEM interval.

## References

[1] Léger D Poursain B Neubauer D Uchiyama M. An international survey of sleeping problems in the general population. *Curr Med Res Opin.* (2008) 24:307–17. 10.1185/030079907X253771 18070379

[2] Colten HR Altevogt BM. Extent and health consequences of chronic sleep loss and sleep disorders. In Colten HR Altevogt BM. *Sleep Disorders and Sleep Deprivation: An Unmet Public Health Problem.* Washington, DC: Institute of Medicine of the National Academies (2006). 55–135. 20669438

[3] Longo LP Johnson B. Addiction: part I. Benzodiazepines–side effects, abuse risk and alternatives. *Am Fam Phys.* (2000) 61:2121–8. 10779253

[4] Greenblatt DJ. Pharmacokinetic determinants of dynamic differences among three benzodiazepine hypnotics. *Arch Gen Psychiatry.* (1989) 46:326. 10.1001/archpsyc.1989.01810040032006 2564763

[5] Roehrs T Roth T. Insomnia pharmacotherapy. *Neurotherapeutics.* (2012) 9:728–38. 10.1007/s13311-012-0148-3 22976558 PMC3480571

[6] Roth T Zorick F Wittig R Roehrs T. Pharmacological and medical considerations in hypnotic use. *Sleep.* (1982) 5:S46–52. 10.1093/sleep/5.suppl_1.S46 6125025

[7] Zammit GK Corser B Doghramji K Fry JM James S Krystal A Sleep and residual sedation after administration of zaleplon, zolpidem, and placebo during experimental middle-of-the-night awakening. *J Clin Sleep Med.* (2006) 02:417–23. 10.5664/jcsm.26657 17557470

[8] Saeed M Naveed M Arif M Kakar MU Manzoor R Abd El-Hack ME Green tea (*Camellia sinensis*) and l-theanine: medicinal values and beneficial applications in humans—a comprehensive review. *Biomed Pharmacother.* (2017) 95:1260–75. 10.1016/j.biopha.2017.09.024 28938517

[9] Sharma E Joshi R Gulati A. l-theanine: an astounding sui generis integrant in tea. *Food Chem.* (2018) 242:601–10. 10.1016/j.foodchem.2017.09.046 29037735

[10] Kakuda T. Neuroprotective effects of theanine and its preventive effects on cognitive dysfunction. *Pharmacol Res.* (2011) 64:162–8. 10.1016/j.phrs.2011.03.010 21477654

[11] Kim S Jo K Hong K-B Han SH Suh HJ. GABA and l-theanine mixture decreases sleep latency and improves NREM sleep. *Pharm Biol.* (2019) 57:64–72. 10.1080/13880209.2018.1557698 30707852 PMC6366437

[12] Kimura R Murata T. Effect of theanine on norepinephrine and serotonin levels in rat brain. *Chem Pharm Bull.* (1986) 34:3053–7. 10.1248/cpb.34.3053 3769108

[13] Yokogoshi H Mochizuki M Saitoh K. Theanine-induced reduction of brain serotonin concentration in rats. *Biosci Biotechnol Biochem.* (1998) 62:816–7. 10.1271/bbb.62.816 9614715

[14] Dodd FL Kennedy DO Riby LM Haskell-Ramsay CF. A double-blind, placebo-controlled study evaluating the effects of caffeine and L-theanine both alone and in combination on cerebral blood flow, cognition and mood. *Psychopharmacology.* (2015) 232:2563–76. 10.1007/s00213-015-3895-0 25761837 PMC4480845

[15] Giles GE Mahoney CR Brunyé TT Taylor HA Kanarek RB. Caffeine and theanine exert opposite effects on attention under emotional arousal. *Can J Physiol Pharmacol.* (2017) 95:93–100. 10.1139/cjpp-2016-0498 28044450

[16] Haskell CF Kennedy DO Milne AL Wesnes KA Scholey AB. The effects of l-theanine, caffeine and their combination on cognition and mood. *Biol Psychol.* (2008) 77:113–22. 10.1016/j.biopsycho.2007.09.008 18006208

[17] Deb S Dutta A Phukan BC Manivasagam T Justin Thenmozhi A Bhattacharya P Neuroprotective attributes of L-theanine, a bioactive amino acid of tea, and its potential role in Parkinson’s disease therapeutics. *Neurochem Int.* (2019) 129:104478. 10.1016/j.neuint.2019.104478 31145971

[18] Egashira N Hayakawa K Osajima M Mishima K Iwasaki K Oishi R Involvement of GABAA receptors in the neuroprotective effect of theanine on focal cerebral ischemia in mice. *J Pharmacol Sci.* (2007) 105:211–4. 10.1254/jphs.SCZ070901 17928735

[19] Kakuda T Nozawa A Sugimoto A Niino H. Inhibition by theanine of binding of [3 H]AMPA, [3 H]Kainate, and [3 H]MDL 105,519 to glutamate receptors. *Biosci Biotechnol Biochem.* (2002) 66:2683–6. 10.1271/bbb.66.2683 12596867

[20] Nishida K Yasuda E Nagasawa K Fujimoto S. Altered levels of oxidation and phospholipase C isozyme expression in the brains of theanine-administered rats. *Biol Pharm Bull.* (2008) 31:857–60. 10.1248/bpb.31.857 18451507

[21] Abbasi B Kimiagar M Sadeghniiat K Shirazi MM Hedayati M Rashidkhani B. The effect of magnesium supplementation on primary insomnia in elderly: a double-blind placebo-controlled clinical trial. *J Res Med Sci.* (2012) 17:1161–9. 23853635 PMC3703169

[22] Li W Yu J Liu Y Huang X Abumaria N Zhu Y Elevation of brain magnesium prevents synaptic loss and reverses cognitive deficits in Alzheimer’s disease mouse model. *Mol Brain.* (2014) 7:65. 10.1186/s13041-014-0065-y 25213836 PMC4172865

[23] Sun Q Weinger JG Mao F Liu G. Regulation of structural and functional synapse density by L-threonate through modulation of intraneuronal magnesium concentration. *Neuropharmacology.* (2016) 108:426–39. 10.1016/j.neuropharm.2016.05.006 27178134

[24] Zhou H Liu G. Regulation of density of functional presynaptic terminals by local energy supply. *Mol Brain.* (2015) 8:42. 10.1186/s13041-015-0132-z 26184109 PMC4504454

[25] Boyle N Lawton C Dye L. The effects of magnesium supplementation on subjective anxiety and stress—a systematic review. *Nutrients.* (2017) 9:429. 10.3390/nu9050429 28445426 PMC5452159

[26] Jacka FN Overland S Stewart R Tell GS Bjelland I Mykletun A. Association between magnesium intake and depression and anxiety in community-dwelling adults: the Hordaland health study. *Aust N Z J Psychiatry.* (2009) 43:45–52. 10.1080/00048670802534408 19085527

[27] Slutsky I Abumaria N Wu L-J Huang C Zhang L Li B Enhancement of learning and memory by elevating brain magnesium. *Neuron.* (2010) 65:165–77. 10.1016/j.neuron.2009.12.026 20152124

[28] ben Zaken S Radomysky Z Koren G. Association between serum magnesium levels and Alzheimer’s disease or mixed dementia patients: a population-based retrospective controlled study. *J Alzheimers Dis Rep.* (2020) 4:399–404. 10.3233/ADR-200220 33163901 PMC7592834

[29] Veronese N Zurlo A Solmi M Luchini C Trevisan C Bano G Magnesium status in Alzheimer’s disease. *Am J Alzheimers Dis Other Demen .* (2016) 31:208–13. 10.1177/1533317515602674 26351088 PMC10852887

[30] le Dieu-Lugon B Dupré N Derambure C Janin F Gonzalez BJ Marret S Effect of neuroprotective magnesium sulfate treatment on brain transcription response to hypoxia ischemia in neonate mice. *Int J Mol Sci.* (2021) 22:4253. 10.3390/ijms22084253 33923910 PMC8074012

[31] Sameshima H Ota A Ikenoue T. Pretreatment with magnesium sulfate protects against hypoxic-ischemic brain injury but postasphyxial treatment worsens brain damage in seven-day-old rats. *Am J Obstetr Gynecol.* (1999) 180:725–30. 10.1016/S0002-9378(99)70279-6 10076154

[32] Terashima T Takido J Yokogoshi H. Time-dependent changes of amino acids in the serum, liver, brain and urine of rats administered with theanine. *Biosci Biotechnol Biochem.* (1999) 63:615–8. 10.1271/bbb.63.615 10361674

[33] Yokogoshi H Kobayashi M Mochizuki M Terashima T. Effect of theanine, r-glutamylethylamide, on brain monoamines and striatal dopamine release in conscious rats. *Neurochem Res.* (1998) 23:667–73. 10.1023/A:1022490806093 9566605

[34] Beker MC Caglayan AB Kelestemur T Caglayan B Yalcin E Yulug B Effects of normobaric oxygen and melatonin on reperfusion injury: role of cerebral microcirculation. *Oncotarget.* (2015) 6:30604–14. 10.18632/oncotarget.5773 26416428 PMC4741555

[35] Iber C Ancoli-Israel S Chesson AL Quan SF. The new sleep scoring manual–the evidence behind the rules. *J Clin Sleep Med.* (2007) 03:107. 10.5664/jcsm.26812

[36] Patel AK Reddy V Araujo JF. Physiology. In: *StatPearls [Internet]*. Treasure Island, FL: StatPearls Publishing. (2021).

[37] Tononi G Cirelli C. Sleep and synaptic homeostasis: a hypothesis. *Brain Res Bull.* (2003) 62:143–50. 10.1016/j.brainresbull.2003.09.004 14638388

[38] Rasch B Born J. About sleep’s role in memory. *Physiol Rev.* (2013) 93:681–766. 10.1152/physrev.00032.2012 23589831 PMC3768102

[39] Gottesmann C. GABA mechanisms and sleep. *Neuroscience.* (2002) 111:231–9. 10.1016/S0306-4522(02)00034-9 11983310

[40] Goetz T Arslan A Wisden W Wulff P. GABAA receptors: structure and function in the basal ganglia. *Prog Brain Res.* (2007) 160:21–41. 10.1016/S0079-6123(06)60003-4 17499107 PMC2648504

[41] Bateson AN. Further potential of the GABA receptor in the treatment of insomnia. *Sleep Med.* (2006) 7:S3–9. 10.1016/j.sleep.2006.03.001

[42] Terunuma M. Diversity of structure and function of GABA B receptors: a complexity of GABA B-mediated signaling. *Proc Jpn Acad Ser B Phys Biol Sci.* (2018) 94:390–411. 10.2183/pjab.94.026 30541966 PMC6374141

[43] Kantrowitz J Citrome L Javitt D. GABAB receptors, schizophrenia and sleep dysfunction. *CNS Drugs.* (2009) 23:681–91. 10.2165/00023210-200923080-00005 19594197 PMC4988234

[44] Kakuda T Nozawa A Unno T Okamura N Okai O. Inhibiting effects of theanine on caffeine stimulation evaluated by EEG in the rat. *Biosci Biotechnol Biochem.* (2000) 64:287–93. 10.1271/bbb.64.287 10737183

[45] Möykkynen T Uusi-Oukari M Heikkilä J Lovinger DM Lüddens H Korpi ER. Magnesium potentiation of the function of native and recombinant GABAA receptors. *Neuroreport.* (2001) 12:2175–9. 10.1097/00001756-200107200-00026 11447329

[46] Bjorvatn B Ursin R. Changes in sleep and wakefulness following 5-HT1A ligands given systemically and locally in different brain regions. *Rev Neurosci.* (1998) 9:265–73. 10.1515/REVNEURO.1998.9.4.265 9886141

[47] Soria-Fregozo C Perez-Vega MI Rodríguez-Landa JF Germán-Ponciano LJ García-Ríos RI Mora-Perez A. *Association of 5-HT1A Receptors with Affective Disorders. Serotonin – A Chemical Messenger Between All Types of Living Cells.* Houston, TX: InTech (2017). 10.5772/intechopen.68975

[48] Parks CL Robinson PS Sibille E Shenk T Toth M. Increased anxiety of mice lacking the serotonin1A receptor. *Proc Natl Acad Sci USA.* (1998) 95:10734–9. 10.1073/pnas.95.18.10734 9724773 PMC27964

[49] Monti JM Jantos H. Dose-dependent effects of the 5-HT 1A receptor agonist 8-OH-DPAT on sleep and wakefulness in the rat. *J Sleep Res.* (1992) 1:169–75. 10.1111/j.1365-2869.1992.tb00033.x 10607047

[50] Nathan PJ Lu K Gray M Oliver C. The neuropharmacology of L-theanine(N-ethyl-L-glutamine): a possible neuroprotective and cognitive enhancing agent. *J Herb Pharmacother.* (2006) 6:21–30. 10.1080/j157v06n02_02 17182482

[51] Ursin R. Serotonin and sleep. *Sleep Med Rev.* (2002) 6:55–67. 10.1053/smrv.2001.0174 12531142

[52] Kesner AJ Lovinger DM. Wake up and smell the dopamine: new mechanisms mediating dopamine activity fluctuations related to sleep and psychostimulant sensitivity. *Neuropsychopharmacology.* (2021) 46:683–4. 10.1038/s41386-020-00903-5 33159169 PMC8027680

[53] Nicoll RAA. Brief history of long-term potentiation. *Neuron.* (2017) 93:281–90. 10.1016/j.neuron.2016.12.015 28103477

[54] Miracca G Soto BA Tossell K Yustos R Vyssotski AL Franks NP Hypothalamic NMDA receptors stabilize NREM sleep and are essential for REM sleep. *bioRxiv* [Preprint]. (2020). 10.1101/2020.10.19.345728

[55] Sebih F Rousset M Bellahouel S Rolland M de Jesus Ferreira MC Guiramand J Characterization of l-theanine excitatory actions on hippocampal neurons: toward the generation of novel N-Methyl-d-aspartate receptor modulators based on its backbone. *ACS Chem Neurosci.* (2017) 8:1724–34. 10.1021/acschemneuro.7b00036 28511005

[56] Sheu S-S Nauduri D Anders MW. Targeting antioxidants to mitochondria: a new therapeutic direction. *Biochim Biophys Acta.* (2006) 1762:256–65. 10.1016/j.bbadis.2005.10.007 16352423

[57] Zeng L Lin L Chen L Xiao W Gong Z. l-theanine ameliorates d-galactose-induced brain damage in rats *via* inhibiting AGE formation and regulating sirtuin1 and BDNF signaling pathways. *Oxid Med Cell Longev.* (2021) 2021:1–13. 10.1155/2021/8850112 34336115 PMC8315880

[58] Kilic U Elibol B Beker M Altug-Tasa B Caglayan AB Beker MC Inflammatory cytokines are in action: brain plasticity and recovery after brain ischemia due to delayed melatonin administration. *J Stroke Cerebrovasc Dis.* (2021) 30:106105. 10.1016/j.jstrokecerebrovasdis.2021.106105 34547676

[59] Muñoz-Castañeda JR Montilla P Padillo FJ Bujalance I Muñ;oz MC Muntané J Role of serotonin in cerebral oxidative stress in rats. *Acta Neurobiol Exp.* (2006) 66:1–6. 16617671 10.55782/ane-2006-1581

[60] Liu Z Khalil RA. Evolving mechanisms of vascular smooth muscle contraction highlight key targets in vascular disease. *Biochem Pharmacol.* (2018) 153:91–122. 10.1016/j.bcp.2018.02.012 29452094 PMC5959760

[61] Siamwala JH Dias PM Majumder S Joshi MK Sinkar VP Banerjee G l-theanine promotes nitric oxide production in endothelial cells through eNOS phosphorylation. *J Nutr Biochem.* (2013) 24:595–605. 10.1016/j.jnutbio.2012.02.016 22819553

[62] Chen X-M Ma Z Kitts DD. Effects of processing method and age of leaves on phytochemical profiles and bioactivity of coffee leaves. *Food Chem.* (2018) 249:143–53. 10.1016/j.foodchem.2017.12.073 29407917

[63] Hwang J-H Kim K-J Ryu S-J Lee B-Y. Caffeine prevents LPS-induced inflammatory responses in RAW264.7 cells and zebrafish. *Chem Biol Interact.* (2016) 248:1–7. 10.1016/j.cbi.2016.01.020 26852703

[64] Kilic E Caglayan B Beker MC. Physiological and pharmacological roles of melatonin in the pathophysiological components of cellular injury after ischemic stroke. *Turk J Med Sci.* (2020) 50:1655–64. 10.3906/sag-2008-32 32962330 PMC7672349

