# Supplementary figures and images for: A Novel Theanine Complex, Mg-L-Theanine Improves Sleep Quality via Regulating Brain Electrochemical Activity
Source: Front Nutr. 2022 Apr 5;9:874254. doi: 10.3389/fnut.2022.874254 (PMC9017334; doi:10.3389/fnut.2022.874254)

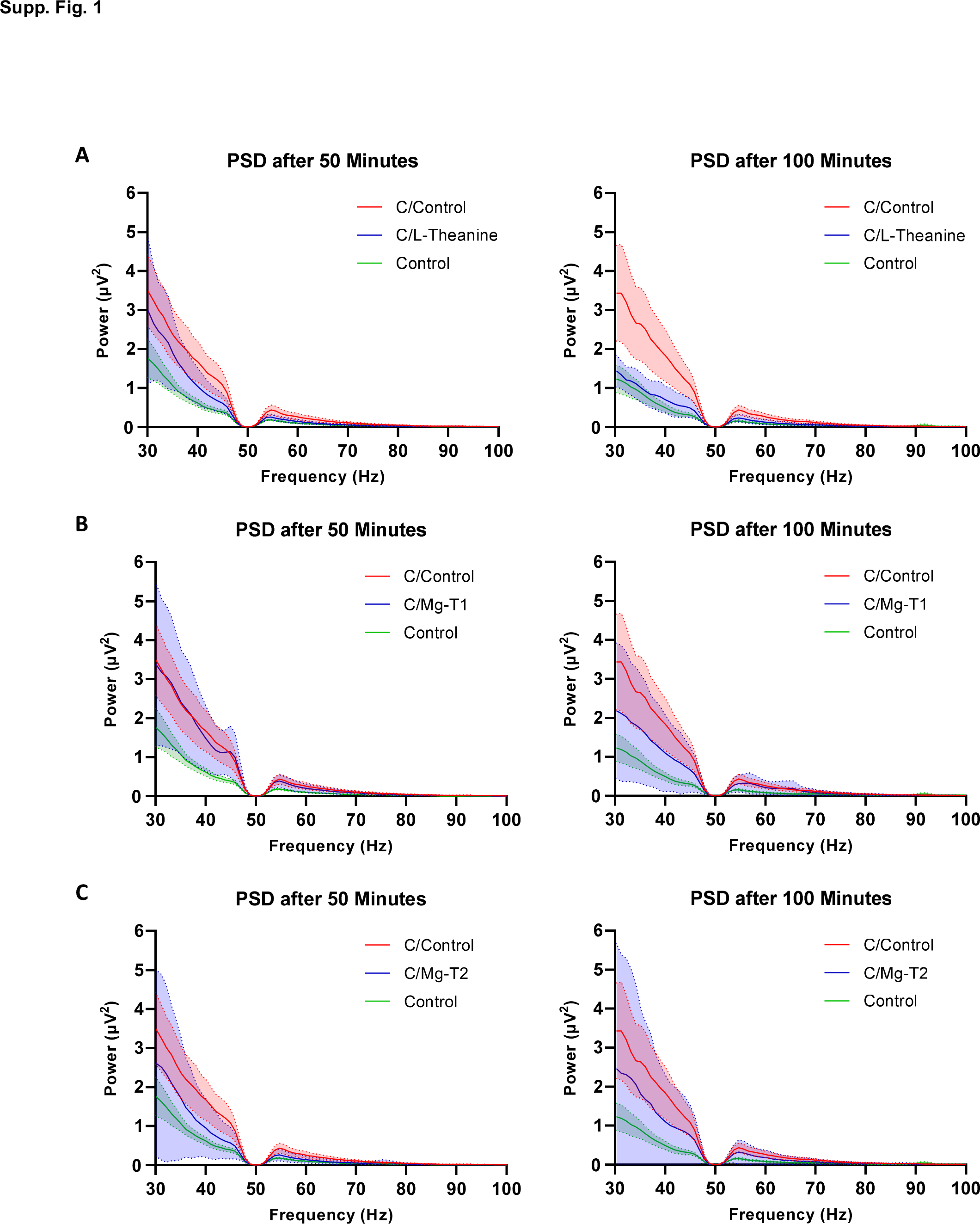

Supplement: Supplementary Figure 1 — Frequency – power graphs of L-theanine (A), Mg-T1 (B), and Mg-T2 (C) at 50th and 100th minutes for 30–100 Hz range. Average of the 1200 FFT (10 min following 50th and 100th minutes) results were calculated for each animal and data were represented as mean ± SEM interval. [file Image_1.TIF]
